# Decompressive craniectomy: A primer for acute care practitioners

**DOI:** 10.1177/17511437241237760

**Published:** 2024-03-24

**Authors:** Peter George Brindley, Mark Sanderson, Dustin Anderson, Cian O’Kelly

**Affiliations:** 1 Department of Critical Care Medicine, University of Alberta, Edmonton, AB, Canada; 2 Division of Neurosurgery, Department of Surgery, University of Alberta, Edmonton, AB, Canada

**Keywords:** Neuro critical care, craniectomy, surgical decompression, ethics, reviews

## Abstract

Decompressive craniectomy (DC) involves surgical removal of the skull that overlies swollen, imperiled, brain. This is done to combat intracranial hypertension and mitigate a vicious cycle of secondary brain injury. If, instead, this pathophysiology goes uninterrupted, it can mean brain herniation and brain stem death. As such, DC can save lives when all else fails. Regardless, it is no panacea and can also “ruin deaths,” and leave patients profoundly disabled. DC is not a new procedure; however, this therapy is increasingly noteworthy due to advances in neurocritical care, alongside ethical concerns. We cover the physiological rationale, the surgical basics, the trial data, and focus on secondary decompression (for refractory intracranial pressure (ICP)) rather than primary decompression (i.e. during evacuation of an intracranial mass). Given that DC should not be undertaken indiscriminately, we conclude by introducing ways in which to discuss DC with families and colleagues. Our goal is to provide a primer and common resource for the multidisciplinary team. We aim to increase not only knowledge but wisdom, prudence, collegiality, and family-focused care.

## Physiology primer

The body’s natural response after injury is to swell. While this is typically part of the healing process, it can be fatal following brain injury. This is because the skull and underlying dura mater restrict expansion; thereby causing high intracranial pressure (ICP) if volume remains constant. Normally, the brain is able to maintain cerebral blood flow (CBF) across a range of cerebral perfusion pressures, via autoregulation.^1^ In excess, however, brain edema, expanding hematomas, and blossoming contusions can exhaust the brain’s compensatory mechanisms. This can create a “vicious cycle” where increased ICP causes decreased cerebral perfusion pressure (CPP) and CBF. In turn, this worsens secondary brain injury which further worsens the likelihood of recovery, and its quality. Alternatively, if ICP can be lowered then we can create a “virtuous cycle,” whereby lower ICP means increased CPP, which increases CBF, and further decreases ICP.

Cerebral pathophysiology was outlined in 1783 by Alexander Monro and expanded upon in 1824 by George Kellie; hence the Monro-Kellie doctrine.^2^ In short, the sum of the volume of intracranial contents – blood, brain, and cerebrospinal fluid (CSF) – must remain equal for the ICP to remain constant. If intracranial contents increase (i.e. through edema or hemorrhage), or if the drainage of blood or CSF is impaired, then the ICP will increase. If the balance of cerebral blood, brain parenchyma, and CSF is markedly altered then it can lead to intractable brain injury. This common pathway can occur after severe traumatic, ischemic, and haemorrhagic brain damage; thus, DC can be considered in all three settings.

DC allows the brain to swell beyond the normal limits of the cranial vault. This increase in intracranial volume can facilitate a decrease in ICP. The goal of therapy, whether medical or surgical, is to optimize the brain’s parenchymal perfusion (inflow) and drainage (outflow).^3^ Accordingly, DC is considered following the failure, or anticipated failure, of cumulative medical therapies such as short-term sedation and normothermia (to reduce cerebral metabolic demands), osmolar and hypertonic therapy (to reduce brain edema), and hyperventilation (to reduce cerebral vasodilation).^4^

Medical therapies have their own risks, particularly if delivered for prolonged periods and at high doses.^5^ As such, DC could offer indirect benefits such as faster anesthetic weaning, shorter hyperventilation, and less time on mechanical support. However, despite the putative physiologic benefits of DC, it carries its own physiologic cautions.^6^ For example, axons may suffer further damage when they stretch abnormally. Similarly, brain tissue can be irreparably damaged by aberrant perfusion, impaired drainage, and altered metabolism. Before we pivot to the surgical specifics, it is worth emphasizing that the goal of DC is not only to save the patient’s life, but also their quality of life. It not merely a dispassionate exercise in improving physiologic parameters and accelerating weaning.

## Surgical primer

Skull surgery -in the form of trephination at least- dates back over 10,000 years.^7^ Strictly speaking, these early surgeries were more like modern burr holes than full decompressions. Regardless, there are various reports as far back as ancient Egypt and Rome, including by Hippocrates and Galen. Skull surgery was further described in 1518 by the Italian Surgeon, da Carpi, and in 1896 by the French Surgeon, Marcotte. In 1901, it was outlined in terms more akin to the modern DC by the Swiss Surgeon, Kocher, and in 1908 by the American Surgeon, Harvey Cushing. In the mid 20th century, DC fell into disrepute given associated mortality rates of 50%–100%. Popularity then steadily increased up to the 2000s, alongside advances in intensive care and neuroimaging.^7^ This has culminated in several randomized controlled trials (RCTs), which are discussed below, and summarized in Tables 1 and 2.

**Table 1.** Summary of decompressive hemicraniectomy studies for malignant infarction.

Publication	Intervention	Population	Outcomes	Conclusions
Juttler, 2007 DESTINY	15 patients in the conservative arm, and 17 patients in the conservative arm plus decompression	Adults 18–60 years old	Mortality at 30 days and 1 year mRS at 6 months and 1 year	Mortality reduced in those patients that underwent decompression plus conservative treatment
Vahedi, 2007 DECIMAL	18 patients in the standard medical therapy arm; 20 patients in the standard medical arm plus decompression	Adults 18–55 years old	Mortality at 6 months and 1 year mRS at 6 months and 1 year	Mortality reduced in those patients that underwent decompression plus medical therapy
Hofmeijer, 2009 HAMLET	32 patients in the standard medical therapy arm; 32 patients in the standard medical arm plus decompression	Adults 18–60 years old	Mortality at 1 year mRS at 1 year Quality of life at 1 year	Mortality reduced in those patients that underwent decompression plus medical therapy
Zhao, 2012	13 patients in the standard medical therapy arm; 16 patients in the standard medical arm plus decompression	Adults 61–80 years old	Mortality at 6 months and 1 year mRS at 6 months and 1 year	Decreased mortality and a more favorable outcome (i.e. a better mRS score). Study included patients older than 60 years
Slezins, 2012	13 patients in the standard medical therapy arm; 11 patients in the standard medical arm plus decompression	Adults 49–81 years old	Mortality at 1 year mRS at 6 months and 1 year	Mortality reduced and more favorable outcomes in patients that underwent decompression. Study, included patients older than 60 years old
Frank, 2014 HeADDFIRST	10 patients in the standard medical therapy arm; 15 patients in the standard medical arm plus decompression	Adults 18–75 years old	Mortality at 6 months mRS at 6 months	No reduction in mortality with decompression
Juttler, 2014 DESTINY II	63 patients in the conservative arm; 49 patients in the conservative arm decompression	Adults 61–82 years old	Mortality at 1 year mRS at 6 months and 1 year Quality of life at 1 year	Survival (without severe disability) higher in patients that underwent decompressive hemicraniectomy
Chua, 2015 HeMMI	13 patients in the standard medical therapy arm; 16 patients in the standard medical therapy arm plus decompression	Adults 18–65 years old	Mortality at 6 months mRS at 6 months	No significant reduction in mortality or favorable outcome at 6 months with decompression

mRS: modified Rankin Scale.

**Table 2.** Summary of decompressive hemicraniectomy studies for traumatic brain injury.

Publication	Intervention	Population	Outcomes	Conclusions
Cooper, 2011 DECRA	155 patients with severe traumatic brain injury, randomized to standard-of care (82) and/or standard of care with decompression (73)	Patients 15–59 years	ICP control; ICP interventions; days in ICU; Extended Glasgow Outcome Scale score, favorable outcome	ICP control and ICU length-of-stay improved following decompression; however, more unfavorable outcomes also observed in this group
Hutchinson, 2016 RESCUEicp	155 patients with severe traumatic brain injury randomized to standard-of care (196) and/or standard of care with decompression (202)	Patients 10–65 years	Extended Glasgow Outcome Scale score at 6 and 12 months; Glasgow Coma Scale score at death or discharge, ICP control	Mortality at 6 months was lower in decompression patients; however, decompression patients also had higher rates of vegetative state
Hutchinson, 2023 RESCUE-ASDH	228 patients underwent craniotomy (bone flap replaced) following acute subdural; 222 had craniectomy (bone not replaced)	Patients >16 years	Extended Glasgow Outcome Score at 6 and 12 months; Glasgow coma score on discharge, ICP control	No significant difference in mortality, disability or vegetative state. Trend toward better outcome, fewer complications, following craniotomy

ICP: intracranial pressure.

Following brain injury, a crani*otomy* (cutting into the skull) is done for hematoma evacuation, and a crani*ectomy* (removing the skull) is done when we anticipate swelling. Of note, bone removal alone is usually insufficient to allow the brain to swell outwards. Accordingly, surgeons frequently include a wide opening of the underlying dura mater. Some surgeons will include an allogenic (“allo” meaning from elsewhere) or autologous (“auto” meaning from yourself) dural-graft to cover the exposed brain. This duraplasty – which is more precisely an expansion duraplasty – allows further decompression and facilitates later replacement of the bone flap (cranioplasty) by avoiding direct adherence of cutaneous tissue to cortical tissue. The excised skull bone can be discarded, or stored in a freezer or, even less commonly, implanted into the patient’s abdominal subcutaneous fat (i.e. marsupialized). If the patient survives, then cranioplasty can be performed weeks to months later, when the swelling has subsided. The stored bone flap can then be replaced or, if it was discarded, the defect can be repaired with a substitute material. Options include pre-fabricated titanium, peak implants, or customized 3-D printed acrylic/composite grafts.

The most common indications for DC are traumatic brain injury (TBI) and stroke/cerebrovascular accident (CVA). Less common is DC following high volume subarachnoid hemorrhage (SAH) and intracranial hemorrhage (ICH).^3^ The most common forms of DC are hemicraniectomy and bifrontal craniectomy, with hemicraniectomy more common. Regardless, the site of injury dictates the site of surgery. TBI and SAH are often diffuse and bilateral, whereas CVA and ICH typically affects one vascular territory or cerebral hemisphere.

The “hemicraniectomy” is more precisely a fronto-temporo-parietal decompressive craniectomy. It starts with a wide curved incision beginning behind or in front of the ear. The scalp flap and temporalis muscle are then deflected to expose the skull. Burr holes are then created and connected to achieve an excision diameter of 12–15 cm. Surgeons take care to avoid exposing the frontal air sinus, and injuring the sagittal sinus and bridging veins. The bifrontal DC involves a bi-coronal skin incision and craniotomy with paracentral burr holes at the level of the coronal suture, and lateral burr holes at the lateral edge of supraorbital ridge. It may also include an extension to a temporal decompression. Given an increased risk of infection, it is better not to open the frontal air sinus. Due to variations in patient anatomy, however, transgression of the frontal air sinus may be required along with exenteration and cranialization (obliteration of the cavity; removal of its contents). Decompression can be further increased with bilateral expansion duraplasty and by dividing the anterior falx. These surgical add-ons increase the risk of a bifrontal craniotomy.

An adequately sized craniectomy is critical, both to lower ICP and to reverse brain shift and herniation. If the craniectomy is too small then trans-calvarial herniation can occur, which effectively traps the exuded brain. This can result in venous kinking, edema, hemorrhage and necrosis. Subsequent cranioplasty is also not benign. It has complications that include – but are not limited to – infection, wound breakdown, hemorrhage, the need for revision, even death^8,9^ Accordingly, the risks of cranioplasty should be part of the consent for a DC.

Our discussion of DC focuses on the aforementioned supratentorial surgeries: hemicraniectomy and bifrontal craniectomy. For the sake of completeness, intensivists should be aware that there are also suboccipital or infratentorial decompressions, aka posterior DC. The pathophysiology is usually spontaneous ICH rather than trauma or ischemic stroke. Swelling in the posterior fossa is often compounded by hydrocephalus which may require an extraventricular drain. This is because the volume of the posterior fossa is small, meaning less tolerance of masses, hemorrhage or swelling. Regardless, the goal decompression is largely similar, namely to relieve pressure (on the brain stem), and minimize herniation (upwards vs tonsillar). Posterior decompression is performed posterior to the cerebellum via a midline incision, from the inion to upper cervical spine. Next, the surgeon exposes the occipital bone by reflecting the lateral muscles. A wide craniectomy is performed and the posterior arch of the atlas often removed. The dura is opened, commonly in a y-shape, along with expansion duraplasty. Of note, cranioplasty is not usually needed for suboccipital/posterior DC, as the surgical defect is small and covered by neck muscles.

## Research trial primer

### Decompression following severe cerebral infarction

Life threatening brain edema occurs in up to 10% of supratentorial strokes.^10^ This usually manifests between day 2 and 5, but can occur sooner. The resultant malignant middle cerebral artery syndrome (MMCAS) is associated with 80% mortality.^10^ Occlusion of this large proximal vessel can cause a cascade of cellular death in a large infarct core, which is surrounded by an at-risk penumbra.^11^ This causes cytotoxic edema, as cellular contents spill out. Later there is also vasogenic edema, as the tight junctions break down between cells and the blood brain barrier becomes permeable.

The cumulative evidence suggests that DC for MMCAS can be worthwhile, albeit far less so in the elderly, or following dominant hemisphere CVAs.^12–17^ Unfortunately, the three studies were small (DESTINY *n* = 32 published in 2007; DECIMAL *n* = 38 published in 2007, and HAMLET *n* = 63 published in 2009). Fortunately, these European studies were similar enough to permit a pooled analysis (*n *= 93).^18^ Overall, there was both an improvement in mortality and the proportion of patients who survived with “good neurologic function,” defined by a “favorable” modified Rankin Scale score (MRSS), namely 0–4.^18^ See Table 3 to better understand the MRSS.

**Table 3.** Modified Rankin Score.

0	No symptoms
1	Symptoms but able to conduct usual activities
2	Unable to carry out all previous activities, but still able to manage own affairs
3	Help is required but still walking without assistance
4	Unable to walk and address bodily needs
5	Bedridden and incontinent
6	Dead

A score of 0–4 is generally considered favorable; 5–6 unfavorable.

These CVA trials highlight the importance of patient selection and early surgery (i.e. within 48 h of deterioration). Enrolled patients were 18–60 years of age, with good pre-morbid neurologic function, and large unilateral MCA strokes but still reactive pupils. The DESTINY-II study investigated older populations (up to 82 years of age) and also showed lower mortality, but less improvement in neurologic function with a significant proportion of patients left severely disabled.^15^

In short, DC for MMCAS is encouraging but no panacea. As with all surgery and intensive care, we need to consider the patient’s age, pre-morbid function, whether dominant or non-dominant cerebral hemisphere, and the availability of prompt surgery and intensive care unit back up. Accordingly, we recommend transferring patients with large hemispheric infarcts before they deteriorate.

### Decompression following severe traumatic brain injury

The DECRA study (full title, “Decompressive Craniectomy in Diffuse Traumatic Brain Injury”) was an RCT investigating bi-fronto-temporo-parietal DC plus duraplasty after severe TBI plus refractory elevated ICP. It was published in 2011 by the *New England Journal of Medicine* and recruitment occurred between 2002 and 2010. This Australasian RCT randomized 155 patients to surgery plus ICU care within 72 h (*n* = 73), versus standard ICU care alone (*n *= 82).^19^

At 6 months, the surgical group had worse neurologic recovery, worse functional outcome, and more severe disability, as measured by the Extended Glasgow Outcome Scale score (Table 4). This was despite the DC group achieving a lower ICP, needing less time on mechanical ventilation, and achieving earlier ICU discharge. Importantly, DECRA excluded those younger than 15 years and older than 59. It also excluded those with mass lesions and intracranial hematoma, those with unreactive pupils, and those post cardiac arrest. This meant that less than 5% of those screened were enrolled (155/3478). Moreover, a single episode of high ICP was sufficient for enrollment. There was a relatively high crossover rate in the standard of care arm, in that 18% received DC. Additionally, some patients in the DC arm likely did not need the surgery.

**Table 4.** Glasgow Outcome Scale Extended (GOSE).

1	Dead
2	Vegetative state
3	Lower severe disability
4	Upper severe disability
5	Lower moderate disability
6	Upper moderate disability
7	Lower good recovery
8	Upper good recovery

A score of 1–4 is considered unfavorable; 5–8 favorable.

Subsequently, the RESCUEicp (full title, “Trial of Decompressive Craniectomy for Traumatic Intracranial Hypertension”) was an RCT published in 2016, also in the *New England Journal of Medicine*.^20^ It included 398 patients who were enrolled between 2004 and 2014, and involved 52 centers in 20 countries. It is generally considered a less restrictive trial than DECRA. This is because RESCUEICP included an age range of 10–65 and there was a choice of DC procedures: either a large unilateral fronto-temporo-parietal craniectomy (i.e. a hemicraniectomy for those with unilateral disease), or a bifrontal craniectomy (for those with diffuse swelling).

One hundred and ninety-six patients received medical therapy alone, and 202 received surgery and medical therapy. The protocol followed escalating (aka “tiered”) medical treatment and was allowed for up to 10 days following injury. Unfortunately, study enrollment took a decade and many centers only enrolled a few patients. In all, DC was associated with lower mortality (26.9% vs 48.9%) but higher rates of being dependent on others for care (21.9% vs 14.4%). There were also more patients in a vegetative state (8.5% vs 2.15%) at 6 months. Rates of moderate disability (23.4% vs 19.7%) and good recovery (4.0% vs 6.9%) were comparable. The long term (24 months) results mirrored the initial analysis. In other words, there was reduced mortality but increased dependency and disability. While the surgical cohort demonstrated some improvement over time, the rate of “good outcome” at 2 years remained similar between groups.^21^

In a further effort to determine whether to decompress, the RESCUE-ASDH (Decompressive Craniectomy vs Craniotomy for Acute Subdural Hematoma) RCT was published in 2023 and randomized acute subdural patients to craniotomy alone (i.e. bone flap replaced) versus DC (i.e. bone flap removed).^22^ Patients were randomized during surgery if the swelling did not preclude bone flap replacement. There was no significant difference in mortality, disability or vegetative outcomes. However, there was an increased incidence of wound complication in the DC arm (12.9% vs 2.9%). Interestingly, this was despite an increased rate of repeat surgery in the craniotomy only arm (14.% vs 6.2% within 2 weeks), thereby emphasizing the risks associated with DC.^22^

In an effort to understand *when* to decompress, Qiu et al.^23^ published a moderately sized RCT (*n* = 74) of unilateral DC within 24 h. They even included patients with herniation. This group reported lower mortality and better functional survival. Lastly, a meta-analysis in 2017 suggested that DC within 36 h was associated with lower mortality and better functional outcomes at 6 months.^24^

### To decompress or not to decompress (following TBI): that is (still) the question

Publication of the DECRA and RescueICP trials led to controversy and debate, not just simple binary answers. The shortcomings of the DECRA trial were summarized by Kitigawa and Bullock (and others) in 2013.^25^ They concluded that DECRA could not be widely generalized because of the exclusive use of bifrontal craniectomy (infrequent in North America, and associated with increased complications), inadequate randomization (an increased proportion in the DC group with fixed pupils and severe CT findings), a highly selected cohort (representing only 1%–2% of severe head injury patients), and inclusion of patients with only a single episode of elevated ICP. It is also difficult to generalize from the Rescue ICP study, given low and uneven ICP increases, and a high crossover rate in the standard arm (over a third; 37%). Despite the best of intentions, the subsequent small studies, and meta-analysis, have also not fully filled in the gaps.

The fourth edition of the Brain Trauma Foundation guidelines, published in 2016, gave a level IIA recommendation that bi-fronto-temporo-parietal DC *should not* be offered after TBI.^26^ The caveat is that these guidelines were published before the RESCUEicp study. In 2020, these guidelines were updated,^27^ with the author’s positing that topics like DC need regular updates or what they called “living guidelines.” The 2020 Brain Trauma Foundation guidelines attempted to provide general guidance despite conflicting and heterogenous evidence. Ultimately, they gave a IIA recommendation *in favor of* late DC for refractory increases in ICP, because it could improve mortality and favorable neurologic outcome. In contrast, they gave an IIA recommendation *against* DC for early refractory ICP elevation, because it is unlikely to improve mortality or favorable neurologic outcomes (as per DECRA). Provocatively, this means that we have general recommendations to operate early in stroke but late in TBI. Regardless, the 2020 TBI guideline update also give a IIA recommendation *against* a bifrontal DC and *for* fronto-temporo-parietal DC. To add to the confusion, the guidelines also provide an IIA recommendation *in favor of* DC for early and later refractory ICP elevation to reduce ICP and shorten ICU length of stay. However, they also acknowledge, rather unsatisfactorily, that “the relationship between these effects and favorable outcome is “uncertain.”^28^

In short, the available literature defies simple answers and indiscriminate application. There are concerns that neither DECRA nor RESCUEicp provided definitive evidence for or against DC following TBI. There is also reticence about undertaking additional trials, given their inherent complexity. At the time of writing, the best advice is nuanced but likely unsatisfactory: evaluate each case, attempt to predict the likely outcome, and explore whether this would be acceptable to that individual patient. In other words, we need to communicate before we operate.

## Multidisciplinary discussions about decompressive craniectomy with families and colleagues

It is challenging to summarize physiology, surgery and research in a single primer. Similarly, we are not suggesting a simplistic one-size-fits all communication strategy. However, because communication is so central to the Intensivist role, and given the importance of multidisciplinary engagement, we offer a few insights. This section also highlights that practitioners need to hone their communication skills every bit as much as their knowledge base or manual dexterity. Communication, decision-making, and uncertainty-management around DC is likely one of the hardest in medicine. As such, efforts are likely to be appreciated and skills are transferable to less dire situations.

Time pressure, uncertainty and even duress is common with DC. As such, doctors may default to curtly telling families that the patient will die without surgery and there is no time to dither. This is not entirely hyperbolic, but can result in families claiming that they were pressured or insufficiently briefed. Alternatively, if we default to being overly dispassionate- *and simply ask families if they want surgery or not*- then we risk appearing uncaring or unsure. Surgeons may feel pressured to intervene, and unable to properly advocate, especially if others have previously suggested surgery. We suggest a middle ground where the family understands that not everyone is a candidate, and that decisions should be individualized to the patient’s age, comorbidities and likelihood of meaningful salvage. Where reasonable, we should partner, and emphasize that there is no perfect/imperfect choice. We should focus on what the patient would say if present, or what most surgeons would recommend at that time for that patient.

We should also focus on the *quality* of survival. This might include explaining how clinicians objectively assess function, whether by GCS, MRSS or GOS-E (Tables 3 and 4). Regardless, we recommend establishing empathic therapeutic partnerships with families and colleagues well before medical therapies are exhausted, and using every opportunity to nourish relationships. This comes from demonstrating that we are capable and caring and available. It also comes from finding time and space for decision making, talking more than once, encouraging reflection, and meeting face-to-face. We also suggest the three-step model popularized by donation physicians.^29,30^ This is based on the three-act play and dates back to at least William Shakespeare. This makes sense when we accept that humans have always been story tellers and our stories need to make sense. It starts with families knowing “what kind of a play this is”: an action-drama where doctors swoop in offering dramatic saves, or a tragedy focusing on limits and comfort.

The three-act approach starts with an act-one that “sets the scene” and “retells the story.” This establishes that “things are bad and may worsen.” Both D-words (namely, death and disability) should be used with substitute decision makers, while emphasizing that we are doing all we can. Act two includes a “telegraphed reveal,” which leads the family toward dramatic news. Then a deliberate interval lets the family reflect, and make-sense. Act three is where decisions are made and we wrap up. Importantly, this theatrical analogy does not mean contrived performances or slavish adherence to a script. Instead, it reminds us that structure helps, and emotions are eternal. Moreover, while DC is ultimately performed by individuals, like a play, it requires engagement from all sides.

## In closing

DC can succeed where all other therapeutic have failed, however, it rarely results in full neurologic recovery. Instead, it is an aggressive intervention to mitigate death and severe disability; which can still happen despite everyone’s best efforts. As such, DC is no panacea, and can feel like an invidious choice of death versus marked disability. Accordingly, each case needs to be reviewed and discussed and deliberated. Stated another way, our verbal dexterity matters as much as manual dexterity or surgical temerity. The literature is incomplete and the only certainty is that neurosurgery, like intensive care, should not be indiscriminately applied. Instead, we need nuance, empathy, and a multidisciplinary partnership that prioritizes the patient’s best interests.
